# The Crosstalk Between B Cells and the Skeletal System During Development, Aging, and in Pathological Conditions

**DOI:** 10.1007/s11914-025-00947-w

**Published:** 2025-12-06

**Authors:** Hanna Terhaar, Brittany Duck, Camden Collins, Emily Grant, Laura Sims Pride, Dan Zhang, Eman Zineldin, Peter D. Burrows, Amjad Javed, Mohamed Khass

**Affiliations:** 1 UAB School of Medicine, Birmingham, AL USA; 2 Department of Microbiology, UAB School of Medicine, Birmingham, AL 35233 USA; 3 Department of Oral and Maxillofacial Surgery, UAB School of Dentistry, Birmingham, AL USA; 4 Department of Endodontics, UAB School of Destistry, Birminghan, AL USA

**Keywords:** B cells, Skeletal system, Development and aging, Autoimmune diseases, Cancer

## Abstract

**Purpose of review:**

In this review, we describe the interaction between B cells and bone during development, aging, and disease.

**Recent findings:**

There is an increased interest in identifying the mechanisms of interaction between immune cells and the skeletal system. This knowledge is critical for understanding the pathology of autoimmune diseases and developing therapeutic interventions. Humoral immunity depends on B cells and their secreted immunoglobulin (antibodies). Earlier studies described B cell influence on the skeletal system, with a major focus on the role of plasma cells and secreted antibodies. The contribution of bone marrow developing B cells to the skeletal system was still poorly studied and represents a gap in our knowledge. This is an active area of investigation in our research group. The crosstalk between B cells and bone starts as early as the commitment of hematopoietic stem cells to the B cell lineage and the differentiation of mesenchymal stem cells to osteoblast progenitors. This crosstalk is active during different developmental stages and continues throughout the life of the individual, especially since both B cells and bone cells share the same developmental niche.

**Summary:**

Bi-directional interaction of developing B cells and osteoblasts, osteoclasts, and chondroblasts ensures their normal development and functional activity. During aging, this interaction is disrupted, leading to disease progression, decreased bone mass, and osteoporosis. A better understanding of B cell-bone interactions will help identify novel immune targets that might provide therapeutic benefit for the elderly and patients.

**Supplementary Information:**

The online version contains supplementary material available at 10.1007/s11914-025-00947-w.

## Introduction

The humoral immune response results in the production of antibodies (immunoglobulins) and cytokines that protect the body against foreign entities. Humoral immunity is mediated by B cells and their progeny, plasma cells, representing the main source of antibody production. The process of antibody generation is tightly regulated during B cell development. Initially, immunoglobulin is expressed as a transmembrane receptor on immature B cells, then secreted at the final stages of B cell maturation in response to antigen. Early B cell development is antigen-independent and begins first in the fetal liver, switching later to the bone marrow (BM). The final stages of B cell maturation are achieved in the peripheral lymphoid organs. Sequential checkpoints during B cell development ensure the production of protective B cells and elimination of pathogenic and autoreactive clones [1]. The final stages of B cell maturation result in the production of plasmablasts and plasma cells that produce soluble antibodies circulating in the bloodstream and in mucosal tissues to protect against foreign substances. Within the BM microenvironment, developing B cells interact with cellular components of the skeletal system, such as Mesenchymal Stem Cells (MSCs), bone-forming osteoblasts, and bone-resorbing osteoclasts. The interaction between these cell types is important for their development and function. Whether this interaction is bi-directional and whether it involves direct physical interaction or is even managed through secreted products, is still an area of active investigation. In this review, we present the known modes of interaction of B cells and bone cells during normal physiological conditions and in disease contexts. A summary of the key findings of research articles cited in the order of reference appearance is provided in Supplementary Table 1.

## Overview of B Cell and Bone Biology

Within the BM microenvironment, different stem cells and cell lineages are located in close contact, which allows for their interaction. Among these cells are developing B cells that originate from Hematopoietic Stem Cells (HSCs) and osteoblasts, myoblasts, chondroblasts, and adipocytes that develop from MSCs. In humans, hematopoiesis is initiated in the yolk sac at 18 days post-conceptional age (PCA). HSCs colonize the fetal liver at 5 weeks PCA, giving rise to developing B cells, which are detected in the fetal liver at 7 weeks [2–4]. The hematopoiesis switch from fetal liver to fetal BM occurs during the second trimester. HSCs begin to be detected in the marrow of long bones at 10–12 weeks PCA. B cell lymphopoiesis is first detectable at 12 weeks PCA [5, 6].

Bone ossification as a result of MSC differentiation into osteoblasts starts at the same time as B cell development, 6–7 weeks PCA [7]. Similar developmental timing and shared developmental location between B cell progenitors and bone cells raise the possibility of close interactions. This interaction continues throughout the life of the individual [8]. During development, B cells influence bone formation through the expression of cytokines and growth factors [9]. Similarly, bone-derived growth factors, such as fibroblast growth factor (FGF-23) and bone-derived osteopontin, influence B cell homeostasis [10, 11]. Dissecting the cellular interactions at different stages of B cell and bone development is essential for understanding the pathogenesis of age-related bone loss and other immune-skeletal-related disorders.

Within the BM, commitment of HSCs into B cell progenitors involves rearrangement of immunoglobulin gene segments by the products of the Recombination Activation Genes (*RAG*), RAG1 and RAG2, which first target variable (Vh), diversity (D), and Joining (Jh) gene segments in the immunoglobulin heavy chain (HC) locus [12]. The first step is D→Jh rearrangement, which occurs in progenitor (pro) B cells, followed by Vh→DJh rearrangement. Successful V(D)Jh recombination results in the production of an IgM Heavy chain (µHC), which demarks precursor (pre) B cells. The functionality of the µHC is tested through its binding to surrogate light chain proteins λ5 and VpreB, to form the preB cell receptor (preBCR), expressed at low levels on the surface of preB cells [13]. Cytokines, such as B cell activating factor (BAFF), IL-4, IL-6, IL-10, IL-7, and IL-21, and transcription factors, such as Pax5, E2A, and Ebf1, are key drivers of this process [14]. Optimum signaling through the preBCR initiates preB cell proliferation, which is followed by light chain (LC) gene rearrangement as a marker of late preB cells. The formation of a complete IgM B cell receptor, composed of the µHC and a κ or λ LC, defines immature B cells. Transcription factors such as BLNK, NF-κB, and Ikzf1 control the development of preB cells into late preB cells and then immature B cells [15–17]. If the immature B cell encounters its cognate antigen in the BM, it is likely a self-antigen, and such self-reactive cells are eliminated or inactivated. The final stages of B cell maturation are completed upon migration of immature B cells from the BM to peripheral lymphoid organs, where, if they encounter cognate antigen, they become activated and differentiate into plasma cells and memory B cells.

The process of bone formation and osteogenesis is achieved through MSC condensation and differentiation into osteoblasts and osteocytes [18]. Both cell types are critical for bone mineralization [19]. Transcription factors such as Runx2 and Osx (osterix) are the main regulators of osteoblast differentiation and maturation, with Runx2 being essential for the commitment of MSCs to osteoblasts and Osx for their maturation into mature and functional bone-forming osteocytes [20, 21]. MSC differentiation into osteoblasts depends on Wnt/β-catenin signaling, which is essential for embryonic development and tissue homeostasis [22]. The process of building bone is coupled with the resorption of unwanted bone. These two opposing physiological functions maintain bone homeostasis. Bone resorption is achieved by osteoclasts, which develop from BM monocytes. Interestingly, osteoclast differentiation is induced through various cytokines and growth factors, including RANKL (receptor activator of nuclear factor kappa-B ligand), secreted in part by osteoblasts. RANKL binds to RANK on the surface of osteoclast precursors to induce their maturation. The RANK decoy receptor OPG (Osteoprotegerin) competes for RANK binding and thus inhibits osteoclast development [23].

Although RANKL signaling is an essential factor in osteoclast regulation, it also influences the survival and development of B cells in the BM [24]. Moreover, osteoblasts secrete the chemokine CXCL12, which supports B cell development [25]. Additionally, osteoblasts secrete IL-7, which is a key cytokine that promotes preB cell survival and differentiation [26]. B cells, on the other hand, secrete pro-inflammatory cytokines such as IL-6, which inhibits osteoblast differentiation [27], while initiating osteoclast differentiation [28].

Identifying the molecular interplay between B cells and bone during development and aging will help us understand their relationship and decipher their roles in aging and autoimmune diseases.

### Crosstalk in Different Skeletal Organs during Development and Growth

Sharing the same niche throughout development, B cells, MSCs, osteoblasts, and osteoclasts interact with one another. MSCs are multipotent cells with characteristics of proliferation, differentiation, and tissue healing. Starting from the second trimester, B cell lymphopoiesis occurs within the BM. Optimal B cell development and proliferation require MSC and osteoblast interactions with developing B cells, which are managed by cell-to-cell crosstalk, cytokine signaling (IL-7, SCF, BMPs, and TGF-β), and extracellular matrix regulation [29]. Simultaneously, MSCs and osteoblasts are influenced by B cells through the secretion of cytokines and inflammatory mediators [30]. The influence of B cells on bone remodeling is mediated through the secretion of RANKL and OPG. Increased B cell expression of RANKL relative to OPG drives bone resorption. This has been observed in osteoporosis, arthritis, and bone immune disorders affecting the vertebrae, femur, tibia, ribs, pelvis, and jaw [27]. Anatomically, long bones harbor large marrow cavities that support lymphopoiesis. Interestingly, the skull contains smaller marrow cavities that harbor B cells with important functions during aging and pregnancy [30]. B cells resident in the skull cavities also play a role in the pathology of stroke, chronic myeloid leukemia, and osteoporosis. Marrow cavities within the skull are supplied with an enriched vasculature that supports hematopoiesis. Moreover, these skull cavities induce regenerative properties that are resilient to aging [30]. Skull marrow cavities also supplement the meninges with B cells, important for immunosurveillance of the central nervous system [31].

## Aging and Immunosenescence

Aging is associated with a functional decline of body organs and the immune system, leading to infection, autoimmunity, and cancer [32]. Similarly, aging results in decreased bone mass, decreased bone mineral density, increased trabecular bone loss, increased cortical bone porosity, and increased fracture risk [33, 34]. During aging, B cell lymphopoiesis is decreased, resulting in decreased B cell numbers and antibody production [35]. This process is triggered by impairment of B cell transcription factors E2A, Pax-5, and STAT5 [36], which affects B cell selection during passage through different selection checkpoints. This can result in the production of autoreactive antibodies, some of which can attack the skeletal system components [37].

Our research has focused on a significant knowledge gap, i.e., the interaction between bone marrow developing B cells and the skeletal system, a research area that has been neglected. Our group was the first to reveal a critical role of precursor (preB) cells in skeletal system homeostasis [38, 39]. We have found that disruption of the preB cell receptor (preBCR) expressed on preB cells caused a significant decrease in both cortical and trabecular bone mass [38]. A critical component of the preBCR is the λ5 protein. Absence of λ5 causes a block in B cell development [40]. Patients with a mutation in the λ5 gene suffer B cell deficiency and agammaglobulinemia [41]. Using our global λ5 knock-out mice, we showed that the absence of λ5 resulted in decreased bone mass, accelerated bone loss, and skeletal aging [39]. The decreased bone mass in λ5-deficient mice seems to be osteoblast-driven since the absence of λ5 only slightly influenced osteoclastogenesis. Human patients with λ5 mutations are extremely rare and usually have residual λ5 protein expression; their bone phenotype has not been well characterized, especially during aging. This is an area of active research in our laboratories.

## B cells, MSCs, and Osteoblasts during Aging

Osteogenesis is dependent on the differentiation of MSCs into functional osteoblasts. Aging induces senescence of MSCs that causes a decrease in their ability to properly differentiate into osteoblasts, which in turn exacerbates bone fragility [42]. Aged MSCs have decreased regenerative potential. This clearly impairs cartilage repair and bone formation, a phenomenon that can be seen in rheumatic diseases such as osteoporosis and arthritis. Decreased MSC regenerative potential predisposes to joint degeneration in osteoarthritis [43].

The interaction between B cells and MSCs promotes osteoblast differentiation [44]. Osteoblasts and their progenitors secrete CXCL-12 and IL-7, which are critical for B cell development [45, 46]. The age-associated decline in B cells may contribute to reduced osteoblastogenesis and impaired bone formation [47].

During aging, there is a decline in developing B cells and MSCs within the bone marrow as well as an increase in pro-inflammatory signaling. This reduction in developing B cell populations contributes to age-related bone loss directly through impaired osteogenic signaling and indirectly through increased inflammatory cytokine production.

## B Cells and Adipocytes during Aging

One of the main features of aged MSCs is their decreased osteogenic activity [48], coupled with increased adipocyte formation that leads to increased marrow adiposity [49]. The contribution of B cells to adipocyte formation during aging remains unknown.

A mouse study of visceral adipose tissue during aging revealed an accumulation of age-associated B cells, suggesting a relationship between B cells, adipocytes, and the adipose tissue environment [50]. The fate decision of MSCs to differentiate into either adipocytes or osteoblasts is mediated through the activity of key transcription factors and signaling cascades, such as Peroxisome Proliferator-Activated Receptor gamma (PPARγ), a key transcription factor that promotes adipogenesis of MSCs, and WNT/beta-catenin signaling, which controls osteoblast formation. During aging, the increased activity of PPARγ relative to WNT/beta-catenin results in a shift toward adipogenesis. This shift influences the BM microenvironment and B cell development [51].

Aging is accompanied by a decrease in early B cell factor-1 (EBF-1) expression that influences B cell development and adipocyte formation [27, 28, 52]. Both adipocytes and B cells secrete RANKL, while B cells express the decoy receptor OPG. The interplay between B cells and adipose tissue can modulate the RANKL-OPG balance and affect bone homeostasis [53].

Aged B cells secrete TNF-α, resulting in impaired B cell development and a decreased number of early B cells (proB cells) [53]. Bone marrow adipocytes have been shown to act as a negative regulator of hematopoiesis, which ultimately influences B cell development [54]. For example, leptin is an adipokine that inhibits B cell apoptosis and stimulates B cell immunosenescence [55, 56]. In summary, aging shifts MSC differentiation toward adipogenesis at the expense of osteoblast differentiation. Adipocytes, in turn, negatively impact B cell development through the production of inflammatory cytokines and alteration of the stromal environment. Further studies are needed to explore the direct influence of adipocytes on B lymphopoiesis.

## B Cells and Osteoclasts during Aging

Aging is accompanied by the increased production of inflammatory cytokines such as IL-1, IL-6, and TNF-α [57]. These cytokines primarily stimulate osteoclast differentiation and function, increasing bone resorption and exacerbating bone loss. Notably, IL-6 and TNF-α can directly stimulate osteoclast formation even with low levels of RANKL expression [58]. Pro-inflammatory cytokines also suppress B cell lymphopoiesis and inhibit MSC differentiation into osteoblasts, shifting their differentiation towards adipogenesis. This, in turn, leads to decreased bone mass and increased marrow adiposity [59].

Thus, aging induces decreased activity of osteoprogenitors, osteoblasts, and osteocytes [60] and increased osteoclastogenesis [61] and adipocyte formation, culminating in bone fragility [53].

### Sex Hormones, Bone Loss, and B Cells During Aging

Major features of aging include loss of bone mass, bone fragility, and higher fracture risk. This can predispose to osteoporosis, particularly among postmenopausal women [62]. Studies of the mechanisms of postmenopausal osteoporosis have focused on the impact of estrogen withdrawal and sex hormones on bone. During menopause, the change in the levels of follicle-stimulating hormone (FSH) increases RANKL expression by T cells, which translates into increased osteoclast development and function, leading to bone loss [63]. Estrogen plays a regulatory role in both osteoclast and osteoblast activity; it supports osteoblast survival while reducing osteoclast development [64]. Estrogen also induces decreased expression of RANKL and contributes to the suppression of pro-inflammatory cytokines, and hence combats bone loss [65]. Interestingly, increased OPG expression by B cells favors bone synthesis over resorption during remodeling [66, 67]. During menopause, estrogen levels are decreased, which leads to increased bone resorption and significant bone loss [68, 69]. Decreased estrogen levels have been associated with increased inflammatory cytokines IL-1, IL-6, TNF-α, IL-17, and RANKL production, all of which promote bone resorption.

## The Role of B Cells in Bone Injury and in Pathological Conditions

### B Cell Role in Bone Healing after Injury

Several studies suggested the contribution of B cells to bone healing after injury [70–73]. The number of B cells recruited to the bone callus influences osteoblast and osteoclast differentiation and thus the healing process [71].

The process of bone healing starts with the recruitment of immune cells to the site of injury to produce an inflammatory hematoma, which develops into a cartilaginous callus that promotes chondrocyte calcification. Osteoblasts and osteochondral progenitors replace the cartilaginous callus with woven bone, which is then remodeled into laminar bone [72, 74]. B cells are recruited to the callus during fracture repair [75]. Within the callus, B cells produce RANKL, CCL3, and TNF-α to promote osteoclastogenesis and bone remodeling [76]. Expression of IL-10 by regulatory B cells (B regs) limits the inflammation at the end stage of healing [77], and B reg dysfunction delays fracture healing [78]. Interestingly, plasma cells recruited to the callus secrete OPG to induce bone formation [79]. Thus, different B cell subsets have different roles during the bone healing process.

### B Cell Cancers and the Bone

Acute lymphoblastic leukemia (B-ALL) is characterized by uncontrolled clonal expansion of progenitor B cells in the bone marrow. B-ALL development is associated with a reduction in the numbers and activity of osteoblasts [80]. B-ALL induces trabecular bone loss, destruction of the growth plate, and decreased adipocyte mass with increased osteoclast number and function [81].

Osteoclasts secrete osteopontin (OPN), a non-collagenous bone matrix that helps local angiogenesis and endosteal adhesion [82], leading to induction of leukemic cell dormancy [83, 84]. The use of OPN-neutralizing antibodies results in rapid B-ALL progression [83].

Alteration in the BM microenvironment is crucial for B-ALL growth and survival. This shifts the normal hematopoietic function to favor leukemia cell expansion and proliferation and enables metastasis of B-ALL into the bone [24].

Multiple myeloma (MM) is another B cell cancer resulting from the proliferation of malignant monoclonal plasma cells in the BM that induce osteolytic lesions [85]. Myeloma cells activate osteoclasts through increased expression of RANKL over OPG, leading to bone loss [86–88]. Similar to B-ALL, MM is associated with higher levels of OPN, which induces extensive osteolytic lesions [89].

### Rheumatoid Arthritis

Rheumatoid arthritis (RA) is a pathological condition characterized by joint inflammation and damage due to the presence of autoantibodies, such as rheumatoid factor (RF) and anti-citrullinated protein antibodies (ACPAs), produced by plasma cells [90]. The autoantibodies form immune complexes that activate osteoclasts and induce bone osteolysis [91]. Proinflammatory cytokines TNF-α, IL-6, and RANKL secreted by B cells induce osteoclast differentiation and bone and joint degradation [92]. Apart from directly promoting osteoclast differentiation, IL-6 and TNF-α enhance the production of the antagonist of the Wnt/β-catenin signaling pathway Dickkopf-1 (Dkk-1) and Sclerostin, which inhibit Wnt signaling and decrease osteoblast differentiation and reduce bone formation [92, 93]. The increased osteoclast activity with decreased osteoblast function augments disruption of bone homeostasis, leading to amplified bone loss.

The mechanism of bone loss during aging mimics what is observed in RA [76], where B cells increase secretion of pro-inflammatory cytokines such as TNF-α and IL-6, in addition to IL-17 secretion by T cells, all of which increase osteoclast activation and loss of bone mass.

### Osteoarthritis

Osteoarthritis (OA) is a degenerative skeletal system disease characterized by progressive deterioration of the joint structure, synovial inflammation, hyperplasia, and fibrosis due to infiltration of leukocytes, including B cells [94–96]. B cell clonal expansion and antibody production have been recognized as contributors to the pathology of OA synovial tissues [97]. The synovial fibroblasts of OA patients induced B cell differentiation and activation and promoted the production of pro-inflammatory cytokines [98]. While the exact mechanism underlying B cell activation is unknown, the levels of the CXCL13 chemokine, which is crucial for attracting B cells, are increased in OA synovium, resulting in B cell recruitment and their chronic activation, leading to increased disease severity [99].

### Defects in B Cells that Lead To Bone Abnormalities during Development and Aging

Changes in either B cells or bone cells induce bi-directional influence during development and aging. In a mouse study, deficiency of PKC-δ expressed by B cells resulted in increased RANKL expression, osteoclast-osteoblast uncoupling, and loss of both trabecular and cortical bone, mimicking the pathology of osteoporosis [100]. B cells secreting IL-10 (Bregs) inhibit osteoclastogenesis, and a decrease in Breg numbers was associated with bone loss in ovariectomized mice [101]. Moreover, Breg numbers normally decrease with aging [102]. This indicates that many B cell factors can contribute to bone loss, either through upregulation of RANKL or even through a decreased number of specific B cell subsets that limit osteoclast activation.

The B cell contribution to periodontitis is well defined. Absence of B cells caused bone loss in a ligature-induced periodontitis mouse model [103]. Receptors expressed on B cells other than RANKL can indirectly influence bone resorption. The binding of erythropoietin to its receptor on B cells increases RANKL expression and increases bone resorption [104]. In a human study of common variable immunodeficiency (CVID) patients, bone loss and osteoporosis have been observed [105]. Interestingly, in this study, the authors claimed that pathological events in CVID patients during childhood lead to later bone mineralization defects upon aging [105].

B lymphocytes secrete TNF-α, which promotes the secretion of MMPs in the synovial fluids and induces the pathogenesis of RA [106]. In a human study of osteosarcoma, infiltration of B cells was correlated with disease progression [107]. Moreover, single-cell sequencing identified two B cell markers associated with an improved clinical outcome [108].

Age-related changes in B cells cause increased expression of inflammatory cytokines that induces a decline in bone mineral density, increases cortical bone porosity, and thinning of the trabecular bone, which leads to osteoporosis and arthritis [109, 110]. Aging induces increased expression of RANKL by B cells, leading to increased bone resorption [111–113]. Aged B cells increased expression of G-CSF, which stimulates osteoclast development [114]. Similarly, aging induces increased collagen cross-linking and bone rigidity, which leads to bone fragility, fracture risk, and osteoporosis [34]. During aging, marrow mesenchymal cells increase expression of PPARγ [115], resulting in increased adipocyte formation relative to osteoblasts. Additionally, PPARγ induces an inhibitory role on osteoblasts through the mTOR pathway [116]. PPARγ promotes cellular senescence through induction of p16^INK4α^ expression [117]. Interestingly, expression of PPARγ by B cells is important for their optimum function. Deficiency of PPARγ in B cells decreases germinal center B cells, plasma cells, and the secreted antigen-specific antibodies [118]. Activation of PPARγ induced apoptosis in preB cells [119], which in turn affects B cell development and indirectly affects bone turnover.

The influence of MSCs on B cells is context-dependent. MSCs induce an inhibitory effect on B cell terminal differentiation and plasma cell function [120]. This inhibition might be mediated through Galectin-9 expression by MSCs [121]. On the other hand, MSCs promote B cell proliferation through BAFF expression [122]. MSCs give rise to different lineages that contribute to bone development and homeostasis. The B cell crosstalk with different cell lineages developed from the MSCs is variable and context- and age-dependent.

B cells express estrogen receptors α and β. The binding of estrogen to its receptor on B cells induces B cell activation and survival through upregulated expression of activation and antiapoptotic markers SHP-1 [123]. Hypoxia Inducible Factor (HIF-1α) signaling in B cells increased RANKL production and osteoclast formation, and its deletion in B cells prevents estrogen deficiency-induced bone loss in mice [124]. Human clinical data revealed a decrease in B cell numbers in the bone marrow of osteoporotic patients. Interestingly, estrogen deficiency in ovariectomized mice increased B cell secretion of G-CSF, which in turn increased osteoclastogenesis and bone resorption [9, 114, 125]. Further studies are needed to delineate the B cell-bone behavior during development, aging, and in the context of diseases.

### Therapeutic Implications

B cells are considered to be a good therapeutic target for hematological cancers and autoimmune diseases. B cell depletion has overlapping effects on bone [126].

The FDA-approved CD20 monoclonal antibody, Rituximab, is used to deplete mature B cells in multiple malignancies and autoimmune diseases such as B-cell non-Hodgkin’s lymphoma (NHL), Chronic lymphocytic leukemia (CLL), Rheumatoid arthritis (RA), Vasculitis, and Pemphigus vulgaris (PV) and other off-label uses [127]. Rituximab’s protective role in bone health has been well-established. It was found that maintenance therapy with rituximab induces a bone-sparing effect, preventing post-induction bone loss in patients with follicular lymphoma [128]. On the other hand, Rituximab had an ambiguous effect on bone mineral density (BMD) in osteoarthritis. In postmenopausal women, Rituximab stabilizes the BMD of spinal vertebrae and forearm bones while it decreases the BMD of femurs [129]. Rituximab has been reported to have jaw and oral complications in patients treated for neurological autoimmune diseases such as multiple sclerosis [130].

The CD20 monoclonal antibody Obinutuzumab, which has been used as an anti-cancer therapy, caused jaw osteonecrosis and dental complications in a patient with follicular non-Hodgkin’s lymphoma [131].

Although several emerging side effects of anti-CD20 therapy have been reported, their underlying mechanisms and optimal treatment strategies remain poorly understood. Further investigation into the relationship between B-cell depletion and site-specific bone effects may help identify the mechanisms by which anti-CD20 therapy influences bone health and guide the development of targeted treatments.

Therapeutic targets of the plasma cell neoplasm multiple myeloma include proteasome inhibitors, immunomodulatory drugs, and bisphosphates and RANKL inhibitors. The proteasome inhibitor, Bortezomib, induces plasma cell apoptosis and improves bone health through induction of bone synthesis and decreasing bone resorption [132]. Its mechanisms of action include induction of Runx2/Cbfa1 and Wnt signaling, suppression of DKK-1, and interactions with the PTH/PTHR1 axis [132].

Multiple myeloma is also treated through targeting B cell Maturation Antigen (BCMA) and CD38, a transmembrane glycoprotein responsible for cell adhesion and signal transduction [133, 134]. The BCMA protein is specifically highly expressed on malignant plasma cells [133, 134]. BCMA has been targeted using CAR T-cell therapies, Bispecific T-cell engagers (BiTEs), and Antibody-drug conjugates (ADC), with no effect on bone mass. Interestingly, the multiple myeloma therapeutic monoclonal antibody, Daratumumab, which targets CD38, showed protective effects on bone and decreased osteoclastogenesis [135–137].

The immunomodulatory drugs thalidomide, lenalidomide and pomalidomide have a protective effect on bone health. Their mechanisms of action include decreasing malignant cell expression of RANKL, MIP-1α, BAFF, and APRIL, inhibiting osteoclast activation and decreased bone loss, and inducing an anti-inflammatory response that protects the bone [137]. Controversially, one in vitro study showed that human osteoblast differentiation was decreased by thalidomide and lenalidomide [138].

### Knowledge Gaps and Future Directions

Despite the substantial progress in elucidating the crosstalk between B cells and the skeletal system, significant knowledge gaps still remain. Notably, the precise mechanisms by which early B cell development influences bone formation and homeostasis are not fully understood. Additionally, while mature B cells have been shown to influence osteoblast and osteoclast lineage commitment within the bone marrow niche, the specific effects of early B cell precursors within the bone marrow microenvironment remain unclear and warrant further investigation. Mechanisms that explain the relationship between declining B cell numbers with increased OPG production in aged bone marrow remain unclear. The molecular cues that coordinate both B cell maturation and bone development in neonatal and adolescent life are yet to be discovered.

In regards to pathological conditions that have been discussed, it is still not fully understood how autoimmune B cell activation drives osteoclastogenesis while simultaneously inhibiting osteoblast function. Future studies should focus on dissecting the bidirectional signaling pathways between early B cells and bone-resident cells, as well as characterizing the cytokine profiles of B cells during disease states and their direct or indirect effects on bone turnover. This information will be pivotal in developing interventions to maintain bone integrity in the context of immune dysregulation and malignancy. The use of the advanced technology of spatial transcriptomics and single cell sequencing, and intravital imaging will certainly identify specific B cell populations and specific RNA and protein targets that are involved in the crosstalk between B cells and osteoblasts, osteoclasts, chondrocytes, myoblasts, adipocytes and mesenchymal stem cells. Dissecting the B cell-bone interaction in different organs such as calvaria, femurs, vertebrae, and ribs, will identify the different contributions of this interaction based on organ type and location. This will require the generation of new global and cell-specific targeted animal models that specifically identify the B cell influence on bone and bone effect on B cell development and function. Humanized mouse models are a valuable tool in this approach. The data obtained from these animal models will be translated into humans through testing patient-derived samples and in vitro organoid systems.

## Conclusion

In summary, there is a dynamic and complex relationship between B cells and bone elements during development, aging, and pathology. Dysregulation of the B cell-bone interaction can induce bone aging and various pathological and autoimmune diseases. During aging, the crosstalk between B cells and the bone microenvironment is altered, ultimately contributing to increased susceptibility to autoimmune disorders and bone-related pathologies. B cell interactions with osteoblasts, adipocytes, and osteoclasts play a pivotal role in modulating immune responses and skeletal health. By understanding how B cells interact with the bone microenvironment, we can identify potential therapeutic targets that are designed to maintain bone integrity while regulating immune responses. Future research should focus on providing additional insights into the key molecular pathways, as well as developing target therapies that play a pivotal role in these interactions, with the goal of improving the quality of life for aged individuals suffering from bone fragility, autoimmune diseases, and cancer.

### Annotated Key References

- Koh BI, Mohanakrishnan V, Jeong H-W, Park H, Kruse K, Choi YJ, et al. Adult skull bone marrow is an expanding and resilient haematopoietic reservoir. Nature. 2024;636(8041):172-81.
  - New advances in studying different skeletal compartments have revealed the novel role of B cells in the marrow cavities in the skull. These B cells are not only important for immune defense but also participate in skull bone regeneration upon injury. It has been found that these B cells rejuvenate the hematopoietic reservoir and continue to be active during aging (Reference 30).
- Brioschi S, Wang W-L, Peng V, Wang M, Shchukina I, Greenberg ZJ, et al. Heterogeneity of meningeal B cells reveals a lymphopoietic niche at the CNS borders. Science. 2021;373(6553):eabf9277.
  - B cells in the skull meninges have been shown to be key hematopoietic cells at the CNS border that help protect the brain and skull tissues from aging (Reference 31).
- Khass M, Rashid H, Burrows PD, Bridges SL, Jr., Javed A, Schroeder HW, Jr. Disruption of the preB Cell Receptor Complex Leads to Decreased Bone Mass. Front Immunol. 2019;10:2063.
  - Relative to mature B cells, our work on early B cells revealed their contribution to bone homeostasis as well.
- Khass M, Rashid H, Burrows PD, Javed A, Schroeder HW. Loss of early B cell protein λ5 decreases bone mass and accelerates skeletal aging. Front Immunol. 2022;13:906649.
  - Absence of the early B cell protein, λ5, caused decreased bone mass and accelerated bone aging. These findings can explain the physiological decrease in bone mass and increase in bone fragility that accompanies aging, which might be contributed in part due to the decrease in the numbers of early B cells and their λ5 expression.

## Supplementary Information

Below is the link to the electronic supplementary material.

Supplementary Material 1(PDF 964 KB)

Supplementary Material 2(DOCX 444 KB)

## Data Availability

No datasets were generated or analysed during the current study.
