# Supplementary material for: The Crosstalk Between B Cells and the Skeletal System During Development, Aging, and in Pathological Conditions
Source: Curr Osteoporos Rep. 2025 Dec 6;23(1):53. doi: 10.1007/s11914-025-00947-w (PMC12680728; doi:10.1007/s11914-025-00947-w)
Supplement: Supplementary file 2 — Supplementary Material 2(DOCX 444 KB) [file 11914_2025_947_MOESM2_ESM.docx]

| **Author (#)** | **Title** | **Type of Study** | **Human/Murine** | **N (patients)** | **Findings** |
| --- | --- | --- | --- | --- | --- |
|  |  |  |  |  |  |
| Popescu, D.M., et al. (2) | Decoding human fetal liver haematopoiesis | Basic science (single-cell sequencing) | Human (fetal) | N/A | Maps hematopoietic development in fetal liver using single-cell RNA-seq. |
| Nuñez, C., et al. (3) | B cells are generated throughout life in humans | Clinical/basic research | Human | dozens | Shows that continuous B cell generation occurs across the human lifespan. |
| Roy, A., et al. (4) | Perturbation of fetal liver hematopoietic stem and progenitor cell development by trisomy 21 | Basic science (developmental biology) | Human (fetal) | N/A | Demonstrates that trisomy 21 alters fetal liver hematopoiesis. |
| O’Byrne, S., et al. (5) | Discovery of a CD10-negative B-progenitor in human fetal life | Basic science (developmental) | Human (fetal) | N/A | Identifies a unique CD10– B cell progenitor with a distinct developmental program. |
| Charbord, P., et al. (6) | Early ontogeny of the human marrow from long bones | Basic science (immunohistochemistry) | Human (fetal) | N/A | Immunohistochemical analysis of early hematopoietic niches in long bones. |
|  |  |  |  |  |  |
|  |  |  |  |  |  |
|  |  |  |  |  |  |
| Fitzpatrick, E.A., et al. (10) | Role of Fibroblast Growth Factor-23 in Innate Immune Responses | Basic science | Murine + Human | N/A | Shows that FGF-23 contributes to innate immune responses. |
|  |  |  |  |  |  |
| Lin, Y.C., et al. (12) | A global network of transcription factors, involving E2A, EBF1 and Foxo1, that orchestrates B cell fate | Basic science (molecular biology) | Human + Murine | N/A | Identifies transcriptional network (E2A, EBF1, Foxo1) that drives B cell lineage commitment. |
|  |  |  |  |  |  |
|  |  |  |  |  |  |
|  |  |  |  |  |  |
| Katerndahl, C.D.S., et al. (16) | Antagonism of B cell enhancer networks by STAT5 drives leukemia and poor patient survival | Basic science / translational | Human + Murine | N/A | Shows that STAT5 disrupts B cell enhancers, driving leukemia progression and poor prognosis. |
|  |  |  |  |  |  |
|  |  |  |  |  |  |
|  |  |  |  |  |  |
|  |  |  |  |  |  |
|  |  |  |  |  |  |
|  |  |  |  |  |  |
|  |  |  |  |  |  |
| Grimaud, E., et al. (22) | Receptor activator of nuclear factor kappaB ligand (RANKL)/osteoprotegerin (OPG) ratio is increased in severe osteolysis | Clinical/Basic Science | Human | 76 | RANKL/OPG ratio significantly increased in patients with severe osteolysis when compared to controls. OPG appears to be protective. |
|  |  |  |  |  |  |
|  |  |  |  |  |  |
| Anguila, H.L., et al. (25) | Osteoblast-specific overexpression of human interleukin-7 rescues the bone mass phenotype of interleukin-7-deficient female mice | Clinical/Basic Science Research | Murine | Unknown | Shows that IL-7 is likely inhibitory for osteoclastogenesis with sex specific differences. |
|  |  |  |  |  |  |
|  |  |  |  |  |  |
|  |  |  |  |  |  |

|  |  |  |  |  |  |  |
| --- | --- | --- | --- | --- | --- | --- |
| Horowitz M.C., et al. (28) | B cells and osteoblast and osteoclast development | Narrative review | Human + Murine | N/A | B cells influence bone remodeling by regulating osteoblast and osteoclast development, thereby linking immune function to skeletal homeostasis. |  |
|  |  |  |  |  |  |  |
| Koh, B.I., et al.(30) | Adult skull bone marrow is an expanding and resilient haematopoietic reservoir | Basic science / translational | Human + Murine | Not specified | Shows that skull marrow provides hematopoietic niches contributing to systemic and CNS immunity. |  |
| Brioschi, S., et al. (31) | Heterogeneity of meningeal B cells reveals a lymphopoietic niche at CNS borders | Basic science (single-cell sequencing) | Human + Murine | Not specified | Meningeal B cells are heterogeneous and form a lymphopoietic niche at CNS borders, supporting local B cell development and immune surveillance. |  |
|  |  |  |  |  |  |  |
|  |  |  |  |  |  |  |
|  |  |  |  |  |  |  |
|  |  |  |  |  |  |  |
|  |  |  |  |  |  |  |
|  |  |  |  |  |  |  |
|  | Riley, R.L., et al.(36) | Deficient B lymphopoiesis in murine senescence: potential roles for dysregulation of E2A, Pax-5… | Basic science | Murine | N/A | Found that senescent mice exhibit deficient B lymphopoiesis, primarily linked to dysregulation of the transcription factors E2A and Pax-5. |
|  |  |  |  |  |  |  |
|  | Khass, M., et al.(38) | Disruption of the preB Cell Receptor Complex Leads to Decreased Bone Mass | Basic science (experimental) | Murine | N/A | A knockout model showing pre-BCR disruption reduces bone mass. |
|  | Khass, M., et al.(39) | Loss of early B cell protein λ5 decreases bone mass and accelerates skeletal aging | Basic science (experimental) | Murine | N/A | A study showing λ5 deficiency leads to bone loss and premature skeletal aging. |
|  | Kitamura, D., et al.(40) | A critical role of lambda 5 protein in B cell development | Basic science (experimental) | Murine | N/A | Foundational study proving λ5 essential for early B cell development. |
|  | Minegishi, Y., et al.(41) | Mutations in the Human λ5/14.1 Gene Result in B Cell Deficiency and Agammaglobulinemia | Clinical research / genetic study | Human | 8 | Rare patient cases showing λ5 mutations cause B cell deficiency and agammaglobulinemia. |
|  |  |  |  |  |  |  |
|  |  |  |  |  |  |  |
|  |  |  |  |  |  |  |
|  | Calvi, M. et al (45) | Osteoblastic cells regulate the hematopoietic stem cell niche | Basic science (experimental) | Murine | N/A | Osteoblastic cells act as a regulatory component of the in vivo haematopoietic stem cell niche, promoting increased haematopoietic stem cell numbers and function via Notch signaling activation. |
|  | Jung, Y. (46) | Regulation of SDF-1 (CXCL 12) production by osteoblasts; a possible mechanisms for stem cell homing. | Basic science (experimental) | Murine | N/A | Osteoblasts regulate SDF-1 (CXCL12) production, which in turn plays a critical role in attracting and retaining haematopoietic stem cells within the bone marrow niche, suggesting a key mechanism for stem cell homing. |
|  | Marusic, A., et al.(47) | Role of B lymphocytes in new bone formation | Basic science (experimental) | Murine | N/A | Demonstrate that B lymphocytes contribute to new bone formation by modulating osteoblast activity and regulating bone remodeling processes. |
|  | Tan, J., et al.(48) | Decreased osteogenesis of adult mesenchymal stem cells by reactive oxygen species under cyclic stretch | Basic science (in vitro, murine) | Murine/in vitro | N/A | Shows oxidative stress reduces osteogenic potential of MSCs, a mechanism in osteoporosis. |
|  | Li, Y., et al.(49) | Nicotinamide phosphoribosyltransferase (Nampt) affects lineage fate of mesenchymal stem cells | Basic science | Human + Murine | N/A | Demonstrates Nampt regulates MSC differentiation, influencing osteogenesis vs adipogenesis. |
|  |  |  |  |  |  |  |
|  |  |  |  |  |  |  |
|  |  |  |  |  |  |  |
|  | Ding, P., et al.(53) | Osteocytes regulate senescence of bone and bone marrow | Basic science | Murine | N/A | Shows osteocytes regulate bone/bone marrow aging and cellular senescence. |
|  | Naveiras, O., et al.(54) | Bone-marrow adipocytes as negative regulators of the haematopoietic microenvironment | Basic science (mouse models) | Murine | N/A | Identifies bone marrow adipocytes as suppressors of hematopoiesis. |
|  | Lam, Q.L.K., et al.(55) | Leptin signaling maintains B-cell homeostasis via induction of Bcl-2 and Cyclin D1 | Basic science | Murine | N/A | Demonstrates that leptin supports B cell survival and proliferation via anti-apoptotic pathways. |
|  | Frasca, D., Blomberg, B.B.(56) | Obesity Accelerates Age Defects in Mouse and Human B Cells | Basic science | Human + Murine | N/A | Shows that obesity worsens age-related immune defects in B cells. |
|  |  |  |  |  |  |  |
|  |  |  |  |  |  |  |
|  |  |  |  |  |  |  |
|  |  |  |  |  |  |  |
|  |  |  |  |  |  |  |
|  | Kim, H.-N., et al. (61) | Osteocyte RANKL is required for cortical bone loss with age and is induced by senescence | Basic science (mouse genetic models) | Murine | N/A | Demonstrates that osteocyte-derived RANKL drives cortical bone loss during aging. |
|  | Imran, M., et al. (62) | Prevalence of Osteoporosis and Associated Risk Factors among Postmenopausal Women | Cross-sectional clinical study | Human | 539 women | Osteoporosis is highly prevalent among postmenopausal women, with key risk factors including advanced age, low body mass index, poor nutrition, and lack of physical activity. |
|  |  |  |  |  |  |  |
|  |  |  |  |  |  |  |
|  |  |  |  |  |  |  |
|  |  |  |  |  |  |  |
|  | Eghbali-Fatourechi, G., et al. (66) | Role of RANK ligand in mediating increased bone resorption in early postmenopausal women | Clinical observational study | Human | 46 women | Demonstrates that RANKL upregulation mediates bone resorption in early menopause. |
|  | Hofbauer, L.C., et al. (67) | Estrogen Stimulates Gene Expression and Protein Production of Osteoprotegerin in Human Osteoblastic Cells | Basic science (cell culture) | Human (in vitro) | N/A | Shows that estrogen increases OPG expression in osteoblasts, protecting against bone resorption. |
|  |  |  |  |  |  |  |
|  |  |  |  |  |  |  |
|  | Roggia, C., et al. (69) | Up-regulation of TNF-producing T cells in bone marrow: Mechanism by which estrogen deficiency induces loss | Basic science (in vivo) | Murine | N/A | Identifies TNF-producing T cells as key mediators of estrogen deficiency–induced bone loss. |
|  | Könnecke, I., et al. (70) | T and B cells participate in bone repair by infiltrating the fracture callus in a two-wave fashion | Basic science (animal fracture model) | Murine | N/A | Demonstrates biphasic infiltration of T and B cells into the fracture callus, contributing to repair. |
|  | Zhang, H., et al. (71) | Single-Cell RNA Sequencing Reveals B Cells Are Important Regulators in Fracture Healing | Basic science (single-cell sequencing) | Murine | N/A | Identifies regulatory B cell subsets involved in fracture healing using single-cell transcriptomics. |
|  |  |  |  |  |  |  |
|  | Sîrbulescu, R.F., et al. (73) | B cells support the repair of injured tissues by adopting MyD88-dependent regulatory functions | Basic science (mechanistic study) | Murine | N/A | Shows that B cells promote tissue repair via MyD88-dependent immunoregulatory phenotype. |
|  |  |  |  |  |  |  |
|  | Molitoris, K.H., et al. (75) | The impact of age and sex on the inflammatory response during bone fracture healing | Basic science (animal study) | Murine | N/A | Age and sex significantly alter inflammatory response and healing outcomes in fracture repair. |
|  |  |  |  |  |  |  |
|  | Yang, S., et al. (77) | Loss of B cell regulatory function is associated with delayed healing in patients with tibia fracture | Clinical observational study | Human | 30–50 patients | Finds that impaired Breg function correlates with delayed tibial fracture healing in humans. |
|  | Sun, G., et al. (78) | Regulatory B cell is critical in bone union process through suppressing proinflammatory cytokines | Basic science (murine fracture model) | Murine | N/A | Bregs promote fracture healing by reducing inflammation and inducing Treg activity. |
|  | Li, Y., et al. (79) | B cells and T cells are critical for the preservation of bone homeostasis and attainment of peak bone mass in vivo | Basic science (knockout mouse study) | Murine | N/A | Shows that B and T cells are essential for achieving peak bone mass and maintaining skeletal homeostasis. |
|  |  |  |  |  |  |  |
|  |  |  |  |  |  |  |
|  |  |  |  |  |  |  |
|  |  |  |  |  |  |  |
|  |  |  |  |  |  |  |
|  |  |  |  |  |  |  |
|  |  |  |  |  |  |  |
|  | |  |  |  |  |  |
|  | Rajakumar, S.A., et al. (81) | B cell acute lymphoblastic leukemia cells mediate RANK-RANKL–dependent bone destruction | Basic science (translational) | Murine + Human | N/A | Shows that ALL cells drive osteoclast-mediated bone destruction through RANK/RANKL signaling. |
|  | Tanaka, Y., et al. (82) | Myeloma Cell–Osteoclast Interaction Enhances Angiogenesis Together with Bone Resorption | Basic science (mechanistic) | Human (in vitro) | N/A | Shows that myeloma–osteoclast crosstalk promotes angiogenesis and osteolysis via VEGF and osteopenia. |
|  | Boyerinas, B., et al. (83) | Adhesion to osteopontin in the bone marrow niche regulates lymphoblastic leukemia cell dormancy | Basic science (cell culture/in vivo) | Human + Murine | N/A | Demonstrates that osteopontin maintains leukemia cell dormancy within the marrow niche. |
|  |  |  |  |  |  |  |
|  |  |  |  |  |  |  |
|  |  |  |  |  |  |  |
|  |  |  |  |  |  |  |
|  |  |  |  |  |  |  |
|  |  |  |  |  |  |  |
|  |  |  |  |  |  |  |
|  |  |  |  |  |  |  |
|  | Delgado-Calle, J., et al. (87) | Bidirectional Notch Signaling and Osteocyte-Derived Factors Promote Tumor Proliferation & Bone Destruction | Basic science (murine and human in vitro) | Human + Murine | N/A | Shows that Notch signaling between osteocytes and myeloma cells enhances proliferation and osteolysis. |
|  | Toscani, D., et al. (88) | The Proteasome Inhibitor Bortezomib Maintains Osteocyte Viability in Multiple Myeloma Patients | Clinical translational study | Human | 37 | Finds that bortezomib preserves osteocyte viability by reducing apoptosis and autophagy. |
|  | Saeki Mima, Y., et al. (89) | Enhanced production of osteopontin in multiple myeloma: clinical and pathogenic implications | Clinical observational study | Human | 81 patients | Shows osteopontin upregulated in MM patients is associated with disease progression and osteolysis. |
|  |  |  |  |  |  |  |
|  | Steffen, U., et al. (91) | How Autoantibodies Regulate Osteoclast Induced Bone Loss in Rheumatoid Arthritis | Basic science (mechanistic study) | Murine + Human | N/A | Demonstrates that autoantibodies promote osteoclastogenesis, leading to bone loss in RA. |
|  | Sun, W., et al. (92) | B cells inhibit bone formation in rheumatoid arthritis by suppressing osteoblast differentiation | Basic science (translational) | Human + Murine | N/A | Shows that B cells suppress osteoblast differentiation, inhibiting bone formation in RA. |
|  |  |  |  |  |  |  |
|  | Felson, D.T., et al. (94) | Synovitis and the risk of knee osteoarthritis: the MOST Study | Prospective cohort study | Human | 1,000+ patients | Finds synovitis strongly associated with progression to symptomatic knee osteoarthritis. |
|  | Krenn, V., et al. (95) | Grading of Chronic Synovitis — A Histopathological Grading System | Methodology / histopathology study | Human | 308  patients | Proposes a standardized histological grading system for chronic synovitis. |
|  |  |  |  |  |  |  |
|  | Da, R.-R., et al. (97) | B Cell Clonal Expansion and Somatic Hypermutation in the Synovial Membrane of Patients with Osteoarthritis | Basic science (molecular immunology) | Human | 5 | Shows clonal expansion and SHM of B cells in OA synovium, indicating adaptive immune involvement. |
|  | Lu, P., et al. (98) | B Cell Activation, Differentiation, and Their Potential Molecular Mechanisms in Osteoarthritic Synovial Tissue | Basic science (molecular pathology) | Human + Murine |  | Demonstrates that activated B cells play a role in the pathogenesis of synovial inflammation in OA |
|  | Shiokawa, S., et al. (99) | Clonal analysis of B cells in the osteoarthritis synovium | Basic science (clonal analysis) | Human | 6 | Identifies oligoclonal B cell populations in OA synovium, suggesting antigen-driven responses. |
|  | Li,S., et al (100). | PKC-delta deficiency in B cells displays osteopenia accompanied with upregulation of RANKL expression and osteoclast-osteoblast uncoupling. | Basic science(molecular immunology) | Murine | 13 | Demonstrates how PKC-δ plays an important role in the interplay between B cells in the immune system and bone cells in the pathogenesis of bone lytic diseases. |
|  | Sapra,L., et al. (101) | Regulatory B Cells (Bregs) Inhibit Osteoclastogenesis and Play a Potential Role in Ameliorating Ovariectomy-Induced Bone Loss. | Basic science (Molecular immunology) | Murine | 24 | Establishes the direct role of regulatory B cells in modulating osteoclastogenesis *in vitro* and suggests that modulations in the percentage of Bregs, along with their reduced potential to produce IL-10, might further exacerbate the observed bone loss in ovx mice. |
|  |  |  |  |  |  |  |
|  | Zeng, W., et al. (103) | B-Cell Deficiency Exacerbates Inflammation and Bone Loss in Ligature-Induced Experimental Periodontitis in Mice. | Basic Sceinces (Molecular immunology) | Murine | 40 | B-cell deficiency exacerbates the inflammation and alveolar bone loss in ligature-induced experimental periodontitis in mice, implicating that B cells may overall play a protective role in the initiation of periodontitis. |
|  | Deshet-Unger, N., et al. (104) | Erythropoietin receptor in B cells plays a role in bone remodeling in mice. | Basic Sciences (Molecular Immunology) | Murine | 10 | Highlights B cells as an important extra-erythropoietic target of EPO-EPO-R signaling and suggests their involvement in the regulation of bone homeostasis and possibly in EPO-stimulated erythropoietic response. |
|  | Baris, S., et al. (105) | Osteoporosis: an ignored complication of CVID. | Clinical Trial | Human | 22 | Osteoporosis appeared as an emerging health problem in patients with CVID, the risk increasing with older age and poorer lung function. Nutritional, biochemical, and immunologic factors appeared to take part in decreased BMD. |
|  |  |  |  |  |  |  |
|  |  |  |  |  |  |  |
|  | Storch, H., et al. (106) | Activated human B cells induce inflammatory fibroblasts with cartilage-destructive properties and become functionally suppressed in return. | Basic science (in vitro + in vivo) | Human + Murine | N/A | Activated B cells drive fibroblasts to adopt inflammatory, cartilage-destructive properties, while fibroblasts in turn suppress B cell function via TGF-β. |
|  | Liu, W., et al. (107) | Exploration of Immune-Related Gene Expression in Osteosarcoma and Association With Outcomes. | Bioinformatic/clinical research | Human | 84 | This genetic association study developed a prognostic modeling tool for osteosarcoma based on IRG expression profiles. |
|  | Zhang, Z., et al. (108) | Identification of B cell marker genes based on single-cell sequencing to establish a prognostic model and identify immune infiltration in osteosarcoma. | Basic science | Human | 6 | Developed a prognostic model for the role of B cells in the development of osteosarcoma. Additionally provides a predictive index and novel therapeutic target for immunotherapy. |
|  |  |  |  |  |  |  |
|  |  |  |  |  |  |  |
|  | Li, Y., et al. (111) | B Cell Production of Both OPG and RANKL is Significantly Increased in Aged Mice. | Basic science (in vivo) | Murine | N/A | Aged mice show decreased bone mass and increased B cell production of OPG and RANKL, with excess RANKL contributing to bone resorption. |
|  | Onal, M., et al. (112) | RANKL protein expression by B lymphocytes contributes to ovariectomy-induced bone loss. | Basic science (in vivo) | Murine | N/A | Loss of RANKL in B cells partially protects against estrogen deficiency–induced cancellous bone loss, showing B cell-derived RANKL is a key mediator. |
|  |  |  |  |  |  |  |
|  | Zhang, Z., et al. (114) | Granulocyte colony stimulating factor (G-CSF) regulates neutrophils infiltration and periodontal tissue destruction in an experimental periodontitis | Basic science | Human + Murine | N/A | Identifies G-CSF as upregulated in inflamed gingiva, driving neutrophil infiltration and pathogenic immune responses in periodontitis, and demonstrates that G-CSF promotes alveolar bone resorption. |
|  |  |  |  |  |  |  |
|  | Sun, H., et al. (116) | Osteoblast-targeted suppression of PPARγ increases osteogenesis through activation of mTOR signaling | Basic science | Murine | N/A | Demonstrates that suppressing PPARγ enhances osteoblast differentiation and reduces adipogenesis and reveals that PPARγ inhibits osteogenesis through suppression of the Akt/mTOR/p70S6K signaling pathway. |
|  | Gan, Q., et al. (117) | PPARγ accelerates cellular senescence by inducing p16^INK4α^ expression in human diploid fibroblasts | Basic science | Murine | N/A | PPARγ promotes cellular senescence in human fibroblasts by upregulating p16INK4α, offering a mechanistic link between PPARγ activity and aging. |
|  | Ramon, S., et al. (118) | Peroxisome proliferator-activated receptor γ B cell-specific-deficient mice have an impaired antibody response | Basic science | Murine | N/A | PPARγ expression in B cells is required for efficient antibody responses by regulating B cell differentiation into plasma cells and memory responses. |
|  | Schlezinger, J.J., et al. (119) | Peroxisome proliferator-activated receptor gamma-mediated NF-kappa B activation and apoptosis in pre-B cells | Basic science (molecular immunology) | Murine | N/A | PPARγ agonists trigger apoptosis through NF-κB–mediated pathways, highlighting the role of PPARγ in B cell development and immune modulation. |
|  | Asari et al.  (120) | Mesenchymal Stem Cells Suppress B-Cell Terminal Differentiation | Experimental in vitro murine study | Murine | NA | Found that MSCs inhibit B-cell terminal differentiation into plasma cells. This suppression occurs without direct contact, as the MSCs act through soluble factors in the culture medium. This blockade is linked to downregulation of Blimp-1, which is a TF driving plasma cell differentiation. |
|  | Fiorina, P, et al.  (121) | Immunomodulatory Function of Bone Marrow-Derived Mesenchymal Stem Cells in Experimental Autoimmune Type 1 Diabetes | Preclinical Experimental Study | Murine | NA | MSCs from different mouse strains were compared: nonobese diabetic (NOD), nonobese resistant mice, and BALB/c mice. They found that MSCs from resistant mouse strains express higher levels of the negative co-stimulatory molecule PD-L1, compared with NOD-MSC. Treating prediabetic NOD mice with allogenic MSCs from resistant mice delayed the onset of diabetes. |
|  | Wang, H., et al.  (122) | Adipogenic Differentiation Alters the Immunoregulatory Property of Mesenchymal Stem Cells through BAFF Secretion. | In vitro cell culture experiments | Murine + Human | NA | When MSCs undergo adipogenic differentiation (Adi), their immunoregulatory properties are changed. Adi-MSCs show increased mRNA and protein expression of BAFF and secrete soluble BAFF. Supernatant from adi-MSCs enhances proliferation and activation of both T and B lymphocytes. When BAFF is blocked by a specific monoclonal Ab, the stimulatory effects from adi-MSCs are greatly reduced. |
|  | Grimaldi, CM., et al.  (123) | Estrogen Alters Thresholds for B Cell Apoptosis and Activation | Experimental in vivo and in vitro study | Murine | NA | It was found that estrogen alters gene expression in B cells, protecting them from apoptosis mediated via the BCR. Additionally, with estrogen exposure, B cells require a stronger antigenic stimulus to undergo apoptosis. |
|  | Meng X., et al.  (124) | Estrogen-Mediated Downregulation of HIF-1a Signaling in B Lymphocytes Influences Postmenopausal Bone Loss | Experimental preclinical | Murine (utilized human B cells/ human gene expression datasets) | NA | Estrogen deficiency leads to increased HIF-1α in B cells and bone loss. Deleting HIF-1a in B cells protects against bone loss, associated with reduced osteoclast numbers/ less bone resorption. This occurs due to estrogen promoting the expression of HSP70 in bone marrow B cells. HSP70 aids the degradation of HIF-1α protein via the proteasomal pathway. |
|  | Nordqvist, J. et al.  (125) | Effects of a Tissue-Selective Estrogen Complex on B Lymphopoiesis and B Cell Function | Experimental preclinical in vivo study | Murine | NA | Estradiol (E2) causes uterine proliferation. When a selective estrogen receptor modulator (bza) is added, the uterine proliferative effects of E2 are blocked. However, E2 + bza did not block the effect of E2 on thymic atrophy; the addition of bza to E2 did not prevent E2-mediated suppression of B cell development, and the E2 + bza treatment did not block the increase in antibody production induced by E2. |
|  |  |  |  |  |  |  |
|  |  |  |  |  |  |  |
|  | Kolomansky, A., et al. (128) | Anti-CD20-Mediated B Cell Depletion Preserves Bone in Lymphoma Patients and Increases Bone Mass in Mice | Clinical + murine translational | Human + Murine | 24 | Shows rituximab preserves bone density in lymphoma patients and increases bone in mice. |
|  | Al Khayyat, S.G., et al. (129) | Bone-sparing effects of rituximab in postmenopausal women with rheumatoid arthritis | Retrospective clinical study | Human | 20 | Finds rituximab preserves bone mass and improves body composition in RA patients. |
|  |  |  |  |  |  |  |
|  | Obermeier, K.T., et al. (131) | Osteonecrosis of the Jaw Associated with Obinutuzumab | Case report | Human | 1 patient | Reports osteonecrosis of the jaw in a lymphoma patient receiving obinutuzumab. |
|  |  |  |  |  |  |  |
|  | Tai, Y.T., et al. (133) | Anti-BCMA antibody-drug conjugate (GSK2857916) selectively kills multiple myeloma cells | Basic science / preclinical | Human in vitro | N/A | Demonstrates the efficacy of GSK2857916 in selectively targeting MM cells. |
|  | Carpenter, R.O., et al. (134) | BCMA as a target for adoptive T-cell therapy in multiple myeloma | Translational preclinical study | Human in vitro | N/A | Shows BCMA is an effective target for adoptive T-cell therapy in MM. |
|  |  |  |  |  |  |  |
|  | Costa, F., et al. (136) | CD38 expression in myeloma bone niche and rationale for anti-CD38 therapy | Basic science + translational | Human + Murine | N/A | Shows CD38 expression promotes osteoclast activity; anti-CD38 therapy inhibits bone destruction. |
|  | Costa, F., Dalla Palma, B., Giuliani, N. (137) | CD38 Expression by Myeloma Cells in Bone Marrow Microenvironment | Translational study (in vitro + ex vivo) | Human | N/A | Demonstrates modulation of CD38 expression by drugs in the MM bone marrow environment. |
|  | Bolomsky, A., et al. (138) | Thalidomide and lenalidomide affect osteoblast differentiation of bone marrow stromal cells | Basic science (cell culture) | Human (in vitro) | N/A | Shows IMiDs influence osteoblast differentiation and may impact bone metabolism. |
